# Anti‐Inflammatory Effects of Hen Egg White Hydrolysate and Its Specific Peptides IS8, PA11, and PK8 on LPS‐Induced Macrophage Inflammation

**DOI:** 10.1002/fsn3.70713

**Published:** 2025-08-03

**Authors:** Ruedeemars Yubolphan, Kajhonpan Phongsiri, Nanthakan Mongkhammee, Jutamas Pidech, Sittiruk Roytrakul, Chonticha Suwattanasophon, Kiattawee Choowongkomon, Sakda Daduang, Watcharee Khunkitti, Nisachon Jangpromma

**Affiliations:** ^1^ Department of Pharmacology, Faculty of Medicine Chiang Mai University Chiang Mai Thailand; ^2^ Protein and Proteomics Research Center for Commercial and Industrial Purposes (ProCCI) Khon Kaen University Khon Kaen Thailand; ^3^ Department of Biochemistry Khon Kaen University Khon Kaen Thailand; ^4^ Functional Proteomics Technology Laboratory National Center for Genetic Engineering and Biotechnology, National Science and Technology Development Agency Pathumthani Thailand; ^5^ Institute of Physiological Chemistry University of Vienna Vienna Austria; ^6^ Department of Biochemistry Kasetsart University Bangkok Thailand; ^7^ Department of Pharmacognosy and Toxicology Khon Kaen University Khon Kaen Thailand; ^8^ Department of Pharmaceutical Technology Khon Kaen University Khon Kaen Thailand

**Keywords:** anti‐inflammation, hydrolysis, LC–MS/MS, nitric oxide, RAW 264.7 macrophages

## Abstract

Egg white hydrolysate (EWH) has garnered significant attention for the therapeutic properties of their bioactive peptides. This study provides a comprehensive peptidomics analysis of EWH, focusing on its anti‐inflammatory potential. Peptides were classified based on their anti‐inflammatory potential (AIP) scores to identify promising candidates. In LPS‐stimulated RAW 264.7 macrophages, specific peptides effectively suppressed nitric oxide (NO) production, a key inflammatory marker. IS8, PA11, and PK8 were identified as the most promising candidates. qRT‐PCR analysis revealed their mechanism of action through modulation of key cytokines, including TNF‐α, IL‐1β, and IL‐10, as well as other mediators such as iNOS and COX‐2. Molecular docking studies provided additional insights into the anti‐inflammatory potential of these peptides. Notably, IS8, PA11, and PK8 exhibited strong binding affinities to the iNOS active site, with IS8 and PK8 achieving the highest docking scores. The peptides engaged in critical interactions within the iNOS binding pocket through hydrogen bonding and π‐π stacking with key residues, suggesting their potential to inhibit iNOS catalytic activity and thereby reduce NO production. These findings highlight the therapeutic potential of EWH‐derived peptides, particularly IS8, PA11, and PK8, as anti‐inflammatory agents through their ability to modulate key inflammatory pathways, with iNOS inhibition as a central mechanism.

## Graphical abstract

Egg white hydrolysate (EWH) peptides IS8, PA11, and PK8 attenuate LPS‐induced inflammation in RAW 264.7 macrophages by reducing NO production and modulating cytokine expression. Molecular docking suggests strong iNOS binding, highlighting their potential as natural anti‐inflammatory agents.

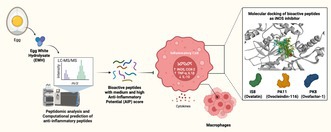

## 1 Introduction

Inflammation, an intrinsic and essential physiological response, plays a dual role in health. Acute inflammation is a protective measure initiated by the body in response to injury or pathogen invasion. In contrast, chronic inflammation can contribute to various diseases, including cardiovascular diseases, cancer, and neurodegenerative disorders (Medzhitov 2010). Mediators of inflammation, such as cytokines, chemokines, and adhesion molecules, orchestrate this immune response, ensuring the containment or elimination of pathogens and facilitating tissue repair (Borish and Steinke 2003). However, when these mediators act unrestrained, the potential for tissue damage and the onset of chronic disease increase (Roe 2021).

The world of nutrition and dietetics has long recognized that diet can have profound physiological implications. Certain foods and their derivatives contain bioactive compounds that can positively or negatively modulate health (Teodoro 2019). There has been an increasing trend to exploit these dietary bioactives, not just for their nutritive value but also for their health‐modifying benefits. Egg whites have garnered significant attention from researchers due to their numerous health benefits. Historically valued for their high‐quality protein content, recent research has highlighted their potential to modulate various health aspects (Dong and Zhang 2021). Egg whites stand as a rich reservoir of proteins, with ovalbumin, ovotransferrin, ovomucoid, ovomucin, and lysozyme being the primary constituents (Abeyrathne et al. 2013). Ovalbumin, the predominant protein (approximately 54% of the total protein), significantly contributes to the unique functional properties of egg whites (Xu et al. 2007). Ovotransferrin, constituting around 12%, exhibits antimicrobial properties (Rathnapala et al. 2021). Ovomucoid, comprising around 11%, functions as a trypsin inhibitor and possesses antimicrobial activity (Abeyrathne et al. 2014). While present in lower concentrations (3.5%), ovomucin has demonstrated potential as a tumor‐suppression agent, inhibiting the growth and spread of certain cancerous cells (Tu et al. 2020). Lastly, lysozyme, also constituting around 3.5%, is widely recognized for its antibacterial properties and its use as a food preservative (Abeyrathne et al. 2013; Levashov et al. 2016). Egg white hydrolysates (EWH) emerge from the alkaline and enzymatic breakdown of these diverse egg white proteins (Li et al. 2022; Wanthong et al. 2024). This process liberates smaller peptides, which are of significant research interest due to their diverse bioactivities. While peptides are the fundamental building blocks of proteins, EWHs exhibit unique biological properties beyond their basic structural role (Zhang et al. 2020). Foremost, their inherent antimicrobial properties position them as potential combatants against a broad spectrum of bacteria, including human pathogens (Levashov et al. 2016; Wanthong et al. 2024). Furthermore, they exhibit strong antioxidative activity, effectively scavenging harmful reactive oxygen species (ROS) and mitigating oxidative damage at the cellular level (Zhou et al. 2022). This antioxidant capacity not only contributes to anti‐aging effects but also protects against oxidative stress‐related diseases (Chen et al. 2023). Egg white‐derived peptides have also demonstrated cytotoxic effects on various human cancer cell lines, hinting at their prospective roles in innovative cancer therapies (Moon et al. 2017). In the context of cardiovascular health, egg white‐derived peptides can inhibit angiotensin‐converting enzyme (ACE), pivotal in regulating blood pressure (Jiayu et al. 2020; Lee et al. 2019). Moreover, egg white‐derived peptides have shown promise in managing metabolic syndrome, including diabetes, by inhibiting enzymes crucial for glucose metabolism (Garces‐Rimon et al. 2016; Jahandideh et al. 2019).

Beyond their multifaceted therapeutic roles, research has further revealed their potential in mitigating inflammation. In examining anti‐inflammatory potential, egg white‐derived peptides stand out in both cellular and in vivo settings. In skeletal muscle cells, these peptides modulate tumor necrosis factor (TNF)‐α‐induced inflammatory signals, highlighting their potential to address inflammation and insulin resistance (Son and Wu 2019). In adipocytes, they exhibit insulin‐sensitizing properties and mitigate inflammatory markers like cyclooxygenase‐2 (COX‐2), offering promise for managing metabolic syndrome complications. In BV2 microglia, egg white‐derived peptides attenuate the overproduction of key inflammatory mediators in a lipopolysaccharide (LPS)‐stimulated environment, providing insights into the therapeutic management of neurodegenerative conditions associated with microglial activation (Choi et al. 2013). In vivo studies, such as employing dextran sodium sulfate (DSS)‐induced colitis models, have shown that administration of egg white‐derived peptides effectively reduces the production of pro‐inflammatory cytokines (TNF‐α, IFN‐γ, and interleukins; ILs), indicating their potential to alleviate gastrointestinal inflammation (Lee et al. 2009). Furthermore, in systemic inflammation models, the peptides demonstrated a capacity to restore and repair damage, as evidenced by their effect on NSAID‐induced duodenal injuries and improvements in intestinal permeability (Playford et al. 2020). In the context of metabolic disorders, prolonged treatment with the peptides not only moderated inflammation‐related complications but also regulated gut microflora, emphasizing their dual role in immune modulation and microbiota balance (Ge et al. 2021). These in vivo outcomes, paired with the aforementioned cellular insights, highlight the promising therapeutic potential of egg white‐derived peptides across a spectrum of inflammatory conditions and metabolic disturbances. However, exploring the mechanism and identifying the specific agents responsible for these observed effects remains essential.

Peptidomics analysis provides a comprehensive overview of the specific peptides present within the sample. However, the identification of these peptides is only the initial step. Functional validation through in vitro and in vivo studies is essential to fully assess their therapeutic potential. Recognizing this, our study employs a peptidomics approach to comprehensively analyze the anti‐inflammatory peptides within EWH. We aim to investigate the anti‐inflammatory potential of these identified peptides and validate their efficacy within the current research context. Through this comprehensive approach, we aim to contribute to the understanding of dietary bioactives, emphasizing the significance of egg white peptides in health promotion and disease prevention.

## 2 Materials and Methods

### 2.1 Egg White Hydrolysate (EWH) Preparation

Hen egg whites (EW) were freshly separated from whole hen eggs purchased from a local market and mixed with 0.4 N KOH in a 1:3 ratio (v/v) for alkaline hydrolysis (Wanthong et al. 2024). The mixture was stirred at 55°C for 2 h. The solution was then autoclaved at 121°C and 15 psi for 2 h. Subsequently, the hydrolysate was filtered through five layers of gauze and neutralized with concentrated hydrochloric acid. The filtrate was centrifuged at 8000 × g for 15 min and collected as egg white hydrolysate (EWH). The EWH was lyophilized and stored at −80°C until use. The lyophilized EW powder was dissolved in sterile distilled water, and the protein concentration was measured using the Bradford protein assay before use in cell‐based experiments.

### 2.2 Peptidomic Analysis and Computational Prediction of Anti‐Inflammatory Peptides From EWH

The peptidomic analysis was conducted at the National Center for Genetic Engineering and Biotechnology, National Science and Technology Development Agency in Thailand. The detailed methodology for protein quantification, including sample preparation, LC–MS/MS analysis, and protein identification parameters, has been previously described (Wanthong et al. 2024). Briefly, the protein concentration was determined using Lowry's assay with BSA as the standard, followed by C18 ZipTip purification and LC–MS/MS analysis (Lowry et al. 1951). Protein quantification was performed using MaxQuant 2.0.3.0 against the Uniprot *Gallus gallus* family database. The potential anti‐inflammatory activity of the peptides was assessed using PreAIP tools as described by Khatun et al. (2019). The tools can be accessed at http://kurata14.bio.kyutech.ac.jp/PreAIP/.

The predicted anti‐inflammatory peptides (AIP) were selected and synthesized using standard Fmoc solid‐phase peptide synthesis. All peptides were purified to a purity of 98% by reverse‐phase high‐performance liquid chromatography (RP‐HPLC), and their molecular weights were confirmed by electrospray ionization mass spectrometry at GL Biochem Ltd. (Shanghai, China).

### 2.3 Functional Analysis of EWH and Its Identified Bioactive Peptides

#### 2.3.1 Nitric Oxide Radical Scavenging Assay

Nitric oxide (NO) levels were determined using the Griess reagent assay (Schmolz et al. 2017). Briefly, EW and EWH (ranging from 15.625–500 μg/mL) were incubated with 20 mM sodium nitroprusside in a 96‐well plate for 2 h at room temperature. Subsequently, the mixture was combined with equal volumes of Griess reagent and allowed to incubate for 10 min. Absorbance was measured at 540 nm using a microplate reader (Viroskan Flash, Thermo Scientific, USA). The NO concentration was determined using sodium nitrite (NaNO_2_) as a standard, and results were reported as % NO reduction.

#### 2.3.2 Cell Culture

RAW 264.7 murine macrophage and normal human dermal fibroblasts (NHDF) cell lines were cultured in Dulbecco's Modified Eagle Medium (DMEM) supplemented with 10% heat‐inactivated fetal bovine serum (FBS) (Sigma‐Aldrich, USA) and 1% antibiotic‐antimycotic solution (Gibco, USA). The cells were maintained at 37°C in a humidified incubator with 5% CO_2_.

Human whole blood was obtained from the Blood Transfusion Center, Faculty of Medicine, Khon Kaen University (ethical approval number HE671415). PBMCs were isolated from whole blood using Ficoll‐Paque PLUS density gradient media (GE Healthcare, Sweden). Blood was diluted 1:1 with PBS and carefully layered onto Ficoll‐Paque PLUS at a 1:1 volume ratio. Centrifugation was performed at 2000 rpm for 35 min at room temperature. After centrifugation, the PBMC layer was carefully transferred to new 15 mL conical tubes. PBMCs were washed three times with 5 mL of 1 × PBS (pH 7.4) by gentle centrifugation at 5000 rpm for 5 min at 4°C. Finally, PBMCs were resuspended in RPMI 1640 medium supplemented with 10% heat‐inactivated fetal bovine serum (Gibco, USA).

#### 2.3.3 Cell Viability Assay

RAW 264.7, NHDF, and PBMCs cells were seeded in 96‐well plates at densities of 2.5 × 10^4^, 1 × 10^4^, and 2 × 10^6^ cells/well, respectively, incubated at 37°C for 24 h, and cultured at 37°C for 24 h. Subsequently, EW, EWH, and its bioactive peptides were serially diluted two‐fold (15.625–500 μg/mL) and added to the cell culture. After 24 h of incubation, the culture medium was replaced with MTT solution (0.5 mg/mL and 100 μL/well), followed by an additional 30 min incubation. The resulting formazan crystals were dissolved in DMSO. Absorbance was then measured at 570 nm using a Viroskan Flash microplate reader (Thermo Scientific, USA). Cell viability was calculated relative to the untreated control cells.

#### 2.3.4 Anti‐Inflammatory Assessment

RAW 264.7 macrophages (2 × 10^4^ cells/well) in 96‐well plates were treated with lipopolysaccharide (LPS; 100 ng/mL) alone or in combination with EW (250 and 500 μg/mL), EWH (250 and 500 μg/mL), or their bioactive peptides (62.5–500 μg/mL) for 24 h. After this period, NO levels in the culture supernatants were determined using the Griess reagent assay, as previously described (Jangpromma et al. 2017). The viability of RAW 264.7 cells was quantified by MTT assay following treatments, using LPS‐stimulated cells as the reference control.

#### 2.3.5 qRT‐PCR: Inflammatory Genes Expression Analysis

To assess the impact on anti‐inflammatory gene expression, RAW 264.7 cells were seeded in 12‐well plates at a density of 2 × 10^5^ cells/well and allowed to incubate overnight. The next day, cells were stimulated with 100 ng/mL of LPS, either alone or combined with bioactive peptides at concentrations of 250 and 500 μg/mL, for 24 h. After this incubation, total RNA was extracted from the RAW 264.7 cells using TRIZOL reagent (Invitrogen, USA) and reverse‐transcribed into cDNA using the Thermo Scientific RevertAid First Strand cDNA Synthesis Kit (Thermo Fisher Scientific, USA). Quantitative real‐time PCR (qPCR) was performed using a LightCycler 480 real‐time PCR system (Roche, Switzerland) with LightCycle SYBR Green I Master mix (Roche, Switzerland). Each 20 μL reaction mixture contained 2 μL of diluted cDNA, 10 μL of SYBR Green Master mix, and 1 μL of each gene‐specific primer (10 μM). The PCR cycling conditions were as follows: initial denaturation at 95°C for 3 min, followed by 40 cycles of denaturation at 95°C for 20 s, annealing at 59°C (as outlined in Table 1) for 20 s, and extension at 72°C for 30 s. Relative gene expression levels were calculated using the comparative 2^−ΔΔCT^ method, with β‐actin as the reference gene.

**TABLE 1:** Primers and qRT‐PCR amplification conditions.

Gene name	Primer sequence (5′ to 3′)		Annealing temperature (°C)	Reference sequence
TNF‐α	Fwd	CCACGCTCTTCTGTCTACTG	59	NM_013693.2
	Rev	ACTTGGTGGTTTGCTACGAC		
IL‐1β	Fwd	AAGCTCTCCACCTCAATGGACAG	59	NM_008361.3
	Rev	CTCAAACTCCACTTTGCTCTTGA		
IL‐10	Fwd	CTATGCTGCCTGCTCTTACTG	59	NM_031168.1
	Rev	CAACCCAAGTAACCCTTAAAGTC		
iNOS	Fwd	TTTCCAGAAGCAGAATGTGACC	59	NM_010927.3
	Rev	AACACCACTTTCACCAAGACTC		
COX‐2	Fwd	GAAATATCAGGTCATTGGTGGAG	59	NM_011198.3
	Rev	GTTTGGAATAGTTGCTCATCAC		
β‐Actin	Fwd	GCTACAGCTTCACCACCACA	59	NM_007393.3
	Rev	AAGGAAGGCTGGAAAAGAGC		

### 2.4 *In Silico* Molecular Docking for Mechanistic Insights of EWH‐Derived Peptides

Gold docking programs (Verdonk et al. 2003), which provide a fitness score to evaluate binding interactions, were utilized in molecular docking experiments. The crystal structure of iNOS bound with the inhibitor AR‐C95791 (PDB ID: 3E7G) was obtained from the Protein Data Bank (https://www.rcsb.org/). The co‐crystallized ligand, AT2 (angiotensin) ligand, was re‐docked into the binding site and used as a comparative standard to evaluate the binding affinity and interaction patterns of the compounds of interest targeting iNOS. The three‐dimensional structures of the potent EWH‐derived peptides, IS8, PA11, and PK8, were constructed and optimized for geometry using Discovery Studio 2021. The docking analyses and visualization of protein‐ligand interaction were performed using BIOVIA Discovery Studio 2021 Visualizer (San Diego, CA, USA).

### 2.5 Statistical Analysis

All experiments were performed in triplicate, and data are expressed as mean ± standard error (SEM). Differences between groups were analyzed using one‐way analysis of variance (ANOVA) followed by a post hoc Tukey test to identify specific group differences. A *p*‐value less than 0.05 was considered statistically significant. All statistical analyses were conducted using GraphPad Prism 9.0 (GraphPad Software, USA).

## 3 Results

### 3.1 Cytotoxic Effects and Anti‐Inflammatory Activity of EWH

**FIGURE 1:**
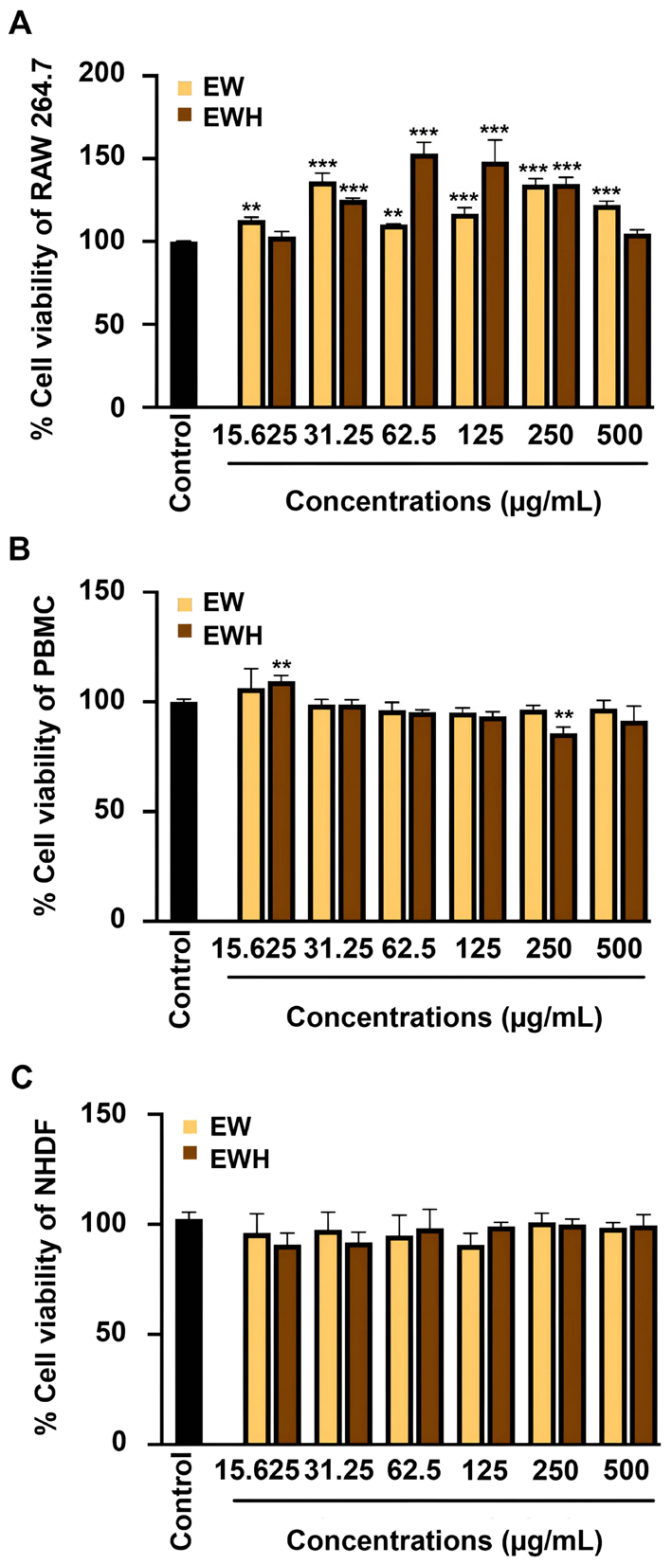
Cell viability assessment of EW and EWH. Cell viability of RAW 264.7 cells (A), PBMCs (B), and NHDFs (C) was assessed after 24 h exposure to EW and EWH at concentrations ranging from 15.625 to 500 μg/mL using the MTT assay. Data are presented as mean ± SEM. *p < 0.05, **p < 0.01, ***p < 0.001 compared to the untreated control group.

**FIGURE 2:**
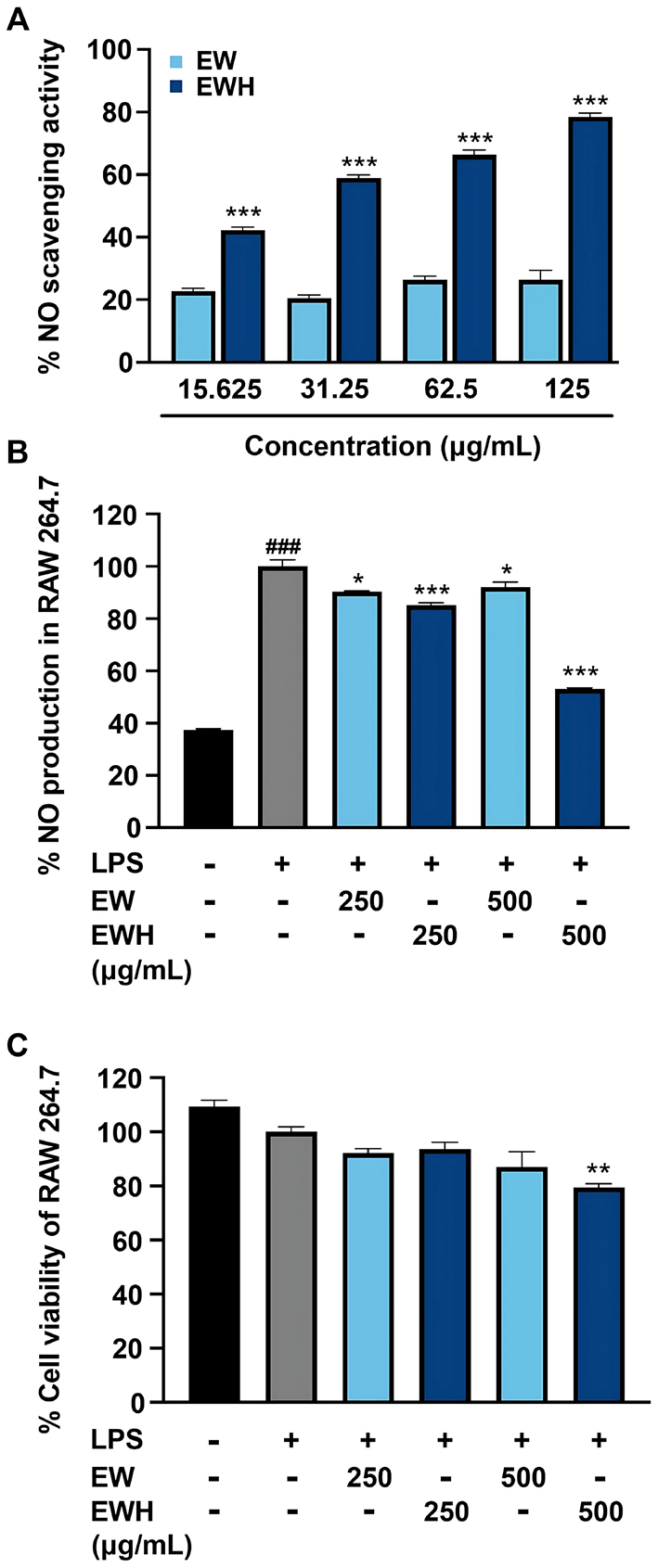
Effects of EW and EWH on nitric oxide (NO) scavenging activity and RAW 264.7 cell viability. (A) NO scavenging activity of EW and EWH at concentrations ranging from 15.625 to 125 μg/mL. (B) NO production levels in LPS‐stimulated RAW 264.7 cells, either untreated or cotreated with EW or EWH at concentrations of 250 and 500 μg/mL. (C) Cell viability of RAW 264.7 cells under LPS‐induced inflammatory conditions, treated or untreated with EW or EWH at concentrations of 250 and 500 μg/mL, as assessed by the MTT assay. Data are presented as mean ± SEM. ^###^ *p* < 0.001 compared to the control group. *p < 0.05, **p < 0.01, ***p < 0.001 compared to the LPS‐treated group.

The cytotoxicity of egg white (EW) and egg white hydrolysate (EWH) was assessed on RAW 264.7 murine macrophage cells, peripheral blood mononuclear cells (PBMCs), and normal human dermal fibroblasts (NHDFs). Across all tested concentrations (15.625–500 μg/mL), neither EWH nor EW exhibited cytotoxicity. Cell viability was maintained comparable to or exceeded the control (100% cell viability) in all three cell lines, with viability consistently above 80% (Figure 1A–C). After confirming the nontoxic nature of EWH, its anti‐inflammatory activity was investigated. Nitric oxide (NO), a critical mediator and inflammation marker (Andrabi et al. 2023), was measured using both chemical assays and cellular models. In the chemical assay, EWH displayed significantly higher NO scavenging activity (42.32% ± 0.86%–78.53% ± 1.15%) compared to EW (22.76% ± 0.89%–26.42% ± 3.06%) at concentrations ranging from 15.625 to 125 μg/mL (Figure 2A). For the cellular models, higher concentrations (250 and 500 μg/mL) were included to evaluate potential protective effects against inflammatory‐induced cytotoxicity, as cellular responses typically require greater concentrations to achieve bioactivity due to variations in uptake, metabolism, and intracellular mechanisms. To evaluate anti‐inflammatory effects in a biological system, RAW 264.7 cells were stimulated with lipopolysaccharides (LPS) to induce an inflammatory response, characterized by increased NO production. EWH treatment (250 and 500 μg/mL) significantly reduced NO levels in a dose‐dependent manner, reaching 85.15% ± 1.56%–53.20% ± 0.62% of the LPS‐treated control group (set as 100% NO production; Figure 2B), highlighting potent anti‐inflammatory potential.

Additionally, the viability of RAW 264.7 cells was assessed under LPS‐induced inflammatory conditions. LPS treatment alone caused a slight reduction in cell viability compared to the untreated control. However, cotreatment with EWH (250 and 500 μg/mL) maintained cell viability at levels comparable to or less toxic than the LPS‐treated group (Figure 2C), indicating minimal additional cytotoxicity under inflammatory conditions.

### 3.2 Peptidomic Analysis and PreAIP Predictions

To elucidate the bioactive components of EWH contributing to these effects, peptidomics analysis was conducted to characterize its peptide composition. Additionally, PreAIP (Predictor of Anti‐inflammatory Peptides) was employed to identify potential anti‐inflammatory peptides within the hydrolysate. These analyses aimed to (i) pinpoint specific peptides underlying the observed bioactivities, (ii) optimize the hydrolysate formulation for enhanced therapeutic efficacy, and (iii) minimize potential toxicity. An in‐depth peptidomic analysis of EWH revealed several peptides derived from *Gallus gallus* (Chicken) proteins (Table 2). The peptides showcased a diversity in mass, from roughly 911.5 to 1024.6 units. The hydrophobic ratios of these peptides spanned between 13% and 63%. They exhibited varying net charges, from −4 to +4. Based on the prediction scores, peptides like IS8, EE9, RE8, and RP8 were classified as high‐confidence anti‐inflammatory peptides (AIP). Meanwhile, peptides such as LE9, PA11, and PK8 were categorized as medium confidence AIP. Key proteins from which these peptides were derived include ovostatin, ovalbumin, vimentin, integrin alpha‐6, ovalbumin‐related protein X, ovocleidin‐116, and ovofactor‐1. These findings underline the potential of these peptides, especially those with a high confidence prediction, to serve as bioactive components in mitigating inflammatory responses.

**TABLE 2:** Peptides originating from EWH through peptidomics, featuring evaluations of their anti‐inflammatory property predictions by PreAIP analysis.

Name	Sequence	Mass (Da)	Protein name	Organism	Hydrophobic ratio	Net charge	PreAIP prediction
IS8	IIDVKMLS	917.5	Ovostatin	*Gallus gallus*	63%	0	High‐confidence AIP
EE9	EVSGLEQLE	1002.5	Ovalbumin	*Gallus gallus*	33%	−3	High‐confidence AIP
RE8	RKLLEGEE	972.5	Vimentin	*Gallus gallus*	25%	−1	High‐confidence AIP
RP8	RVTNLGRP	911.5	Integrin alpha‐6	*Gallus gallus*	25%	2	High confidence AIP
LE9	LPDEVSDLE	1015.5	Ovalbumin‐related protein X	*Gallus gallus*	33%	−4	Medium‐confidence AIP
PA11	PAPSTGGRIVA	1024.6	Ovocleidin‐116	*Gallus gallus*	36%	1	Medium‐confidence AIP
PK8	PEKKAKKK	955.6	Ovofactor‐1	*Gallus gallus*	13%	4	Medium‐confidence AIP

### 3.3 Evaluation of Bioactive Peptides on RAW 264.7 Cells Under LPS Stimulation

Peptidomic analysis of the EWH identified seven peptides with medium and high‐confidence predictions for anti‐inflammatory properties, as predicted by PreAIP. These peptides were synthesized using the Fmoc solid‐phase methodology at GL Biochem Ltd. (Shanghai, China). Following synthesis, the peptides were purified to 98% purity through reverse‐phase high‐performance liquid chromatography (HPLC). Subsequently, their structural integrity and molecular composition were confirmed through electrospray ionization‐mass spectrometry. To further understand the potential anti‐inflammatory properties of the identified bioactive peptides, their effect on NO production and cell viability was evaluated in LPS‐stimulated RAW 264.7 macrophages. LPS stimulation of RAW 264.7 cells induced an increase in NO production.

**FIGURE 3:**
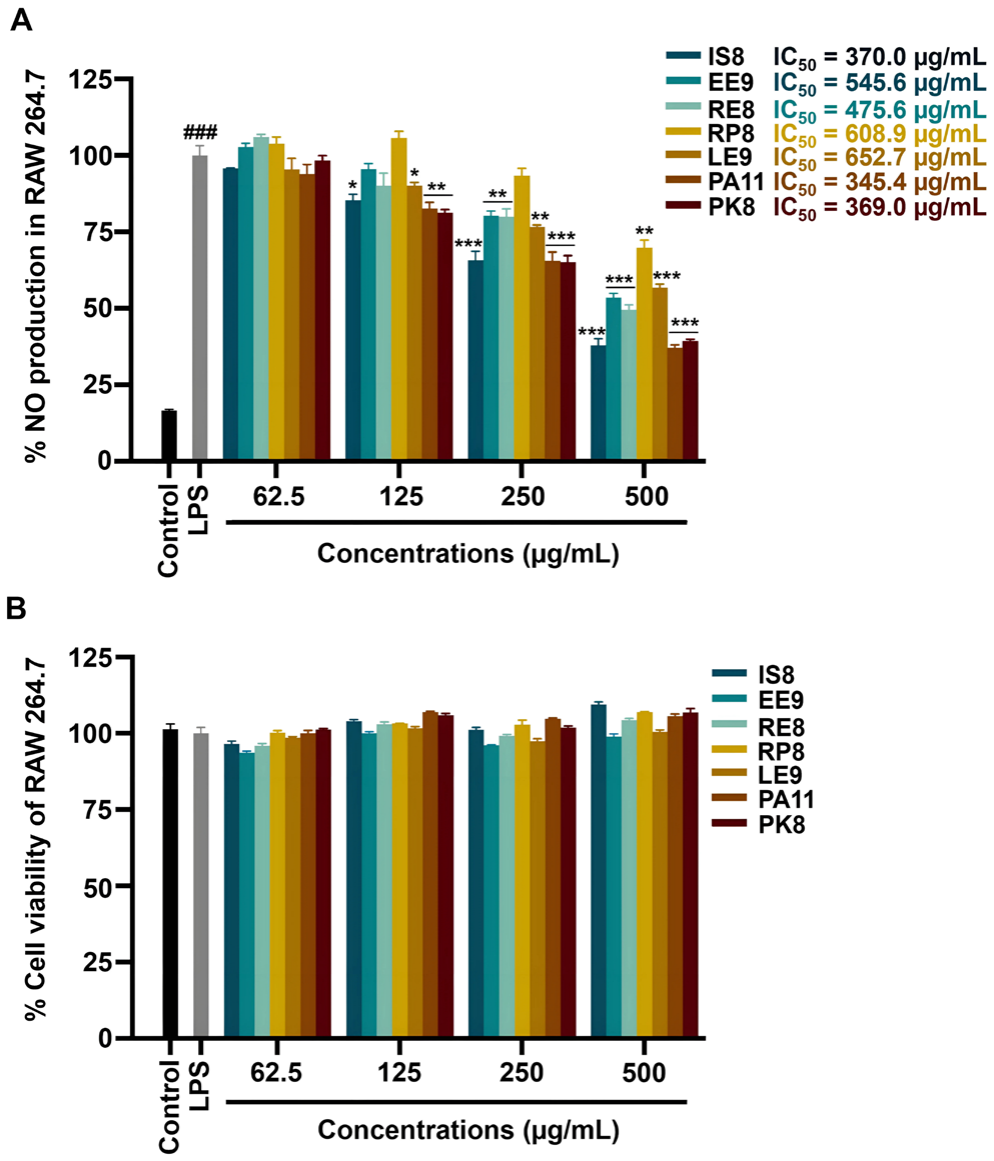
Anti‐inflammatory effects of bioactive peptides on LPS‐stimulated RAW 264.7 cells. (A) Nitric oxide (NO) production in LPS‐stimulated RAW 264.7 cells after treatment with bioactive peptides (IS8, EE9, RE8, RP8, LE9, PA11, and PK8). IC_50_ values for NO inhibition by each peptide were determined using GraphPad Prism 9.0. (B) Cell viability of RAW 264.7 cells under LPS‐induced inflammatory conditions, treated or untreated with bioactive peptides, as assessed by the MTT assay. Data are presented as mean ± SEM. ^###^ *p* < 0.001 compared to control. *p < 0.05, **p < 0.01, ***p < 0.001 compared to LPS‐treated group.

When the selected bioactive peptides (IS8, EE9, RE8, RP8, LE9, PA11, and PK8) were introduced to these cells at concentrations ranging from 62.5 to 500 μg/mL, a dose‐dependent decline in NO production was observed (Figure 3A). This reduction in NO levels highlighted the anti‐inflammatory potential of these peptides, with the IC_50_ values for NO inhibition ranging from 345.4 to 652.7 μg/mL. Among these, IS8 (IC_50_ = 370.0 μg/mL), PA11 (IC_50_ = 345.4 μg/mL), and PK8 (IC_50_ = 369.0 μg/mL) exhibited the lowest IC_50_ values, suggesting they are more potent anti‐inflammatory peptides (Figure 3A). Cell viability was assessed using the MTT assay to evaluate the effects of the peptides on LPS‐stimulated RAW 264.7 cells. LPS treatment alone did not significantly alter cell viability compared to the untreated control group. Similarly, cells cotreated with LPS and the peptides (at doses ranging from 62.5 to 500 μg/mL) maintained their viability, comparable to those of the LPS‐treated control group (Figure 3B). These findings suggest that the peptides did not exhibit cytotoxic effects under inflammatory conditions, and further, showed promise in modulating inflammation by reducing NO production without significant toxicity under LPS‐stimulated conditions. This finding provides a critical foundation for determining safe and effective doses for further anti‐inflammatory evaluations. Finally, the three peptides with the lowest IC_50_ values (IS8, PA11, and PK8) were further investigated to evaluate their anti‐inflammatory mechanisms using qRT‐PCR.

### 3.4 Effects of Bioactive Peptides From EWH on Pro‐Inflammatory Mediators in LPS‐Activated Macrophages

**FIGURE 4:**
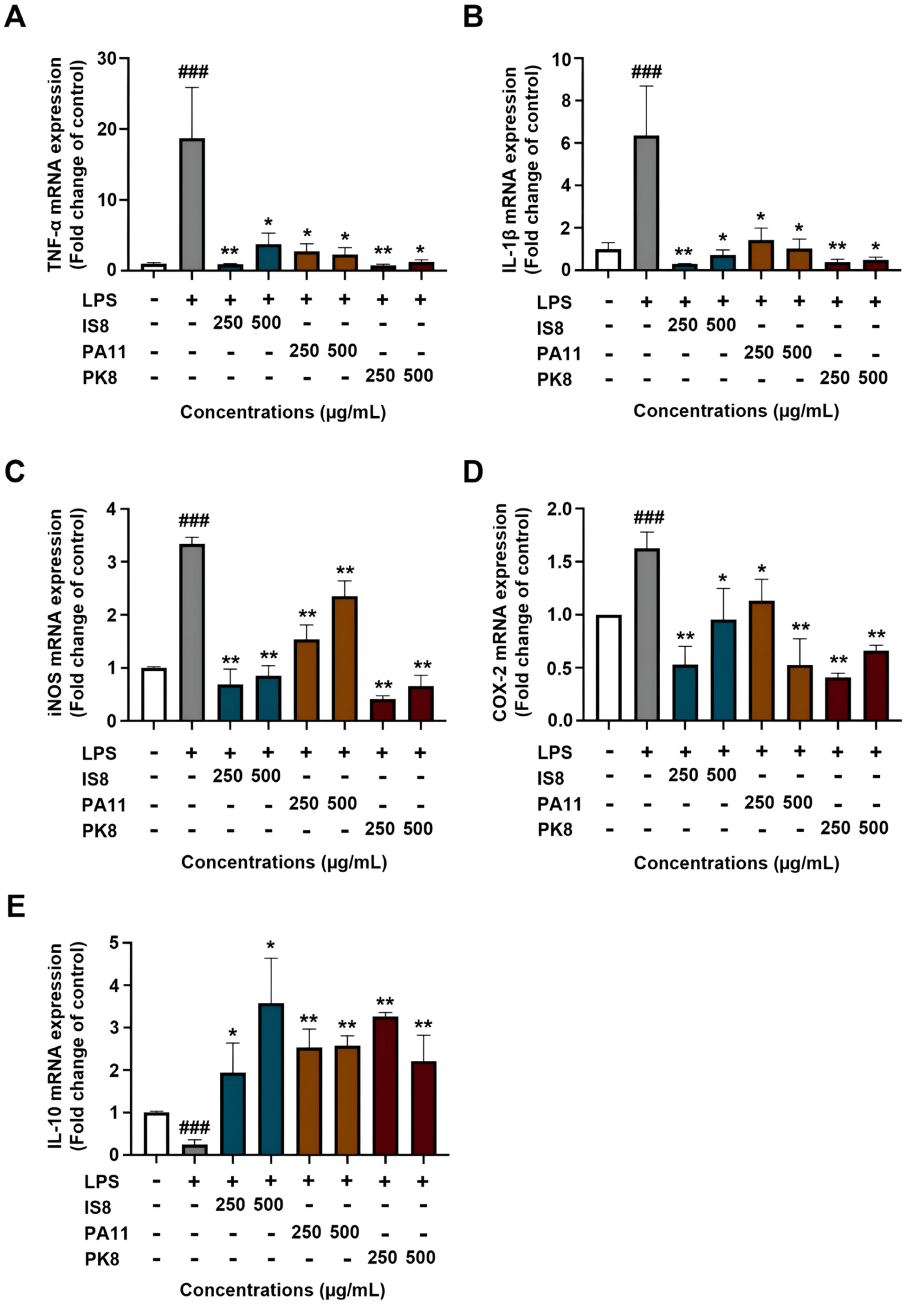
Effects of EWH‐derived bioactive peptides on pro‐inflammatory mediators in LPS‐stimulated macrophages. RAW 264.7 cells were prepared and subjected to the LPS challenge. Along with the LPS stimulation, cells were also treated with each bioactive peptide at concentrations of 250 and 500 μg/mL for 24 h. qRT‐PCR was employed to assess the expression of genes associated with proinflammatory mediators: mRNA levels of (A) TNF‐α, (B) IL‐1β, (C) iNOS, (D) COX‐2, and (E) IL‐10. Data are presented as mean ± SEM. ^###^ *p* < 0.001 compared to control. *p < 0.05, **p < 0.01, ***p < 0.001 compared to LPS group.

The role of pro‐inflammatory mediators, including cytokines such as TNF‐α, IL‐1β, and enzymes like iNOS and COX‐2, is central to the inflammation process. Meanwhile, IL‐10 serves as a counteracting anti‐inflammatory cytokine. The bioactive peptides from EWH, specifically IS8, PA11, and PK8, were examined for their potential effects on these mediators. RAW 264.7 cells were plated and challenged with LPS. Along with LPS stimulation, cells were also treated with each bioactive peptide at concentrations of 250 and 500 μg/mL for a duration of 24 h. As depicted in Figure 4A–D, LPS stimulation significantly upregulated the mRNA expression of pro‐inflammatory mediators: TNF‐α, IL‐1β, iNOS, and COX‐2. However, in the presence of bioactive peptides, there was a noticeable reduction in the expression of these mediators (Figure 4A–D). On the contrary, the expression of the anti‐inflammatory cytokine IL‐10 was enhanced in the peptide‐treated groups compared to the LPS‐only treated cells (Figure 4E). This pattern suggests that these bioactive peptides potentially modulate the inflammatory response by suppressing pro‐inflammatory mediators and promoting the expression of anti‐inflammatory agents.

### 3.5 *In Silico* Evaluation of Molecular Interaction Between Inducible Nitric Oxide Synthase (iNOS) and Bioactive Peptides From EWH

Molecular docking studies were conducted to investigate the interaction between iNOS, a key enzyme involved in inflammation, and bioactive peptides derived from EWH. The co‐crystallized ligand, AT2, was redocked into the iNOS binding site as a positive control, serving as a reference for binding interactions. AT2 inhibits iNOS activity by interfering with the conversion of L‐arginine to NO, thereby reducing inflammation (Isaksson et al. 2020). Its binding pose provides a reliable benchmark for evaluating the binding efficiency of other potential inhibitors or therapeutic peptides. The docking scores for AT2 and the peptides IS8, PA11, and PK8, calculated using the Gold docking program, are presented in Table 3. The docking results revealed that all peptides had higher docking scores than AT2, indicating stronger binding affinities. Specifically, the scores were as follows: AT2 (57.51), IS8 (107.55), PA11 (76.77), and PK8 (108.77). These findings suggest that the bioactive peptides derived from EWH exhibit a higher potential for interaction with the iNOS active site compared to AT2.

**TABLE 3:** The binding scores of AT2 and bioactive peptides derived from EWH calculated by the Gold docking program.

Ligand name	Binding score
AT2	57.51
IS8	107.55
PA11	76.77
PK8	108.77

**FIGURE 5:**
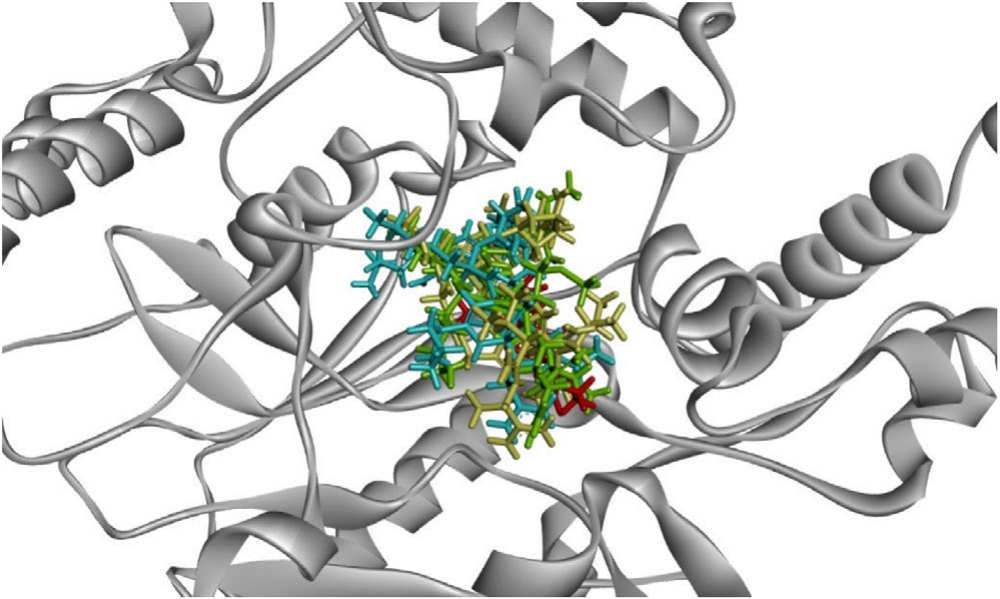
The superimposed best docking poses of AT2 (red) and bioactive peptides derived from EWH: IS8 (green), PA11 (yellow), and PK8 (blue) with the inducible nitric oxide synthase (iNOS) structure.

**FIGURE 6:**
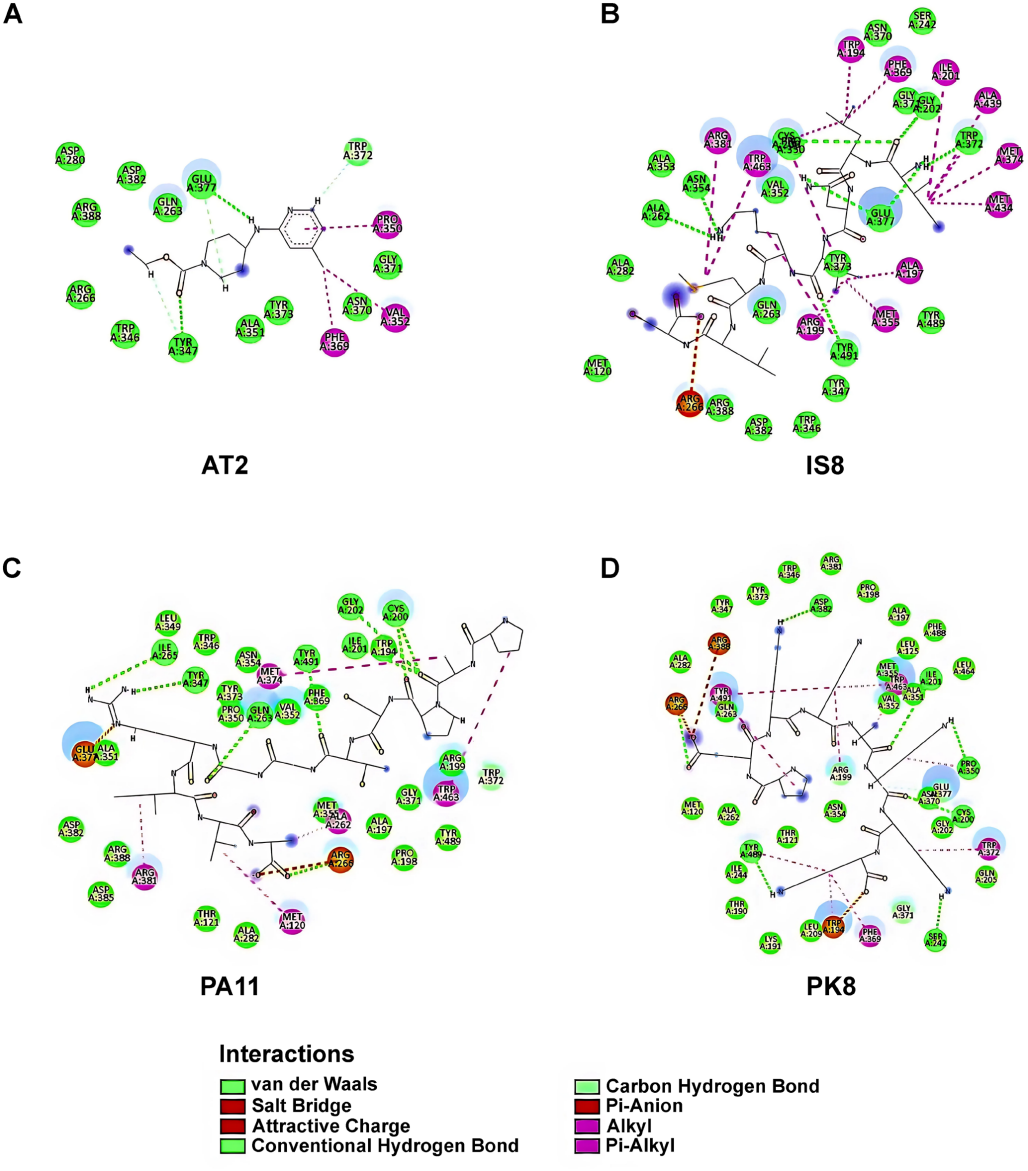
Two‐dimensional diagrams of the interactions of AT2 (A) and bioactive peptides derived from EWH: IS8 (B), PA11 (C), and PK8 (D) with the iNOS structure.

All ligands, including AT2 and the peptides, were observed to share the same binding pocket on iNOS, as illustrated in Figure 5. The interaction analysis (Figure 6) revealed that the ligands not only engaged in van der Waals interactions but also formed hydrogen bonds, hydrophobic interactions, and π‐π stacking interactions with residues in the binding pocket. IS8 and PK8 demonstrated nearly identical docking scores, which were the highest among the peptides. Both formed multiple hydrogen bonds and π‐π stacking interactions with active site residues, enhancing their stability within the binding pocket. PA11, with an intermediate binding score, displayed a combination of hydrophobic interactions and hydrogen bonding, contributing to its interaction with the iNOS active site.

## 4 Discussion

The complex interplay between diet and health has revealed numerous bioactive compounds with profound physiological implications. Within this spectrum, egg whites have emerged as a treasure trove, with their peptides holding significant promise (Abeyrathne et al. 2013; Dong and Zhang 2021). This study delves deep into the peptidomic intricacies of egg white hydrolysate (EWH), revealing a dynamic profile that appears poised to modulate LPS‐induced inflammation in the RAW 264.7 macrophage model. Our data, illustrating the prowess of EWH against LPS‐induced inflammation in RAW 264.7 macrophages, echo prior studies that have sought to unravel the therapeutic depths of egg white derivatives. Choi et al. (2013) were pivotal in this exploration, elucidating the potential of egg white derivatives to inhibit pro‐inflammatory mediators in microglia, such as iNOS, COX‐2, and various cytokines (TNF‐α and IL‐1β). Concurrently, Jahandideh et al. (2017) provided insights into how egg white digests can adeptly modulate cytokine secretion and sensitize insulin signaling in adipocytes; this strengthens the case for the potential role of EWH in controlling inflammatory cascades. Son and Wu (2019) also shed light on the remarkable ability of EWH to reverse insulin resistance associated with TNF‐α in skeletal muscle cells (Son and Wu 2019).

Peptidomics offers a comprehensive investigation into the functional attributes of peptide compositions (Arena et al. 2020). Through our analysis, we aimed to decode the peptidomic complexities of EWH and pinpoint peptides with potent anti‐inflammatory roles. A major highlight from our peptidomic results was the identification of peptides endowed with amino acid sequences indicative of anti‐inflammatory capabilities. As emphasized by Abeyrathne et al. (2013), the presence of acidic amino acids (aspartic acid and glutamic acid) and alkaline types (arginine and lysine) enhances the radical scavenging potential of these peptides (Abeyrathne et al. 2013). This composition could underpin the potent anti‐inflammatory mechanisms exhibited by EWH. The profile of hydrophobic amino acids adds further intricacy. Prior studies highlight the significance of amino acids like alanine, leucine, tyrosine, valine, and phenylalanine (Kovacs‐Nolan et al. 2005). The criticality of these residues goes beyond mere structure, as they are pivotal in protecting lipid structures. Their potential role in donating protons to lipid radicals offers a unique dimension to the broader anti‐inflammatory narrative. On closer examination, seven peptides stood out, primarily due to their elevated predicted anti‐inflammatory potential (PreAIP) (Khatun et al. 2019). For instance, peptide IS8 (IIDVKMLS) originating from ovostatin exhibited a high confidence AIP. Ovostatin, a potent protease inhibitor found in egg whites, plays a significant role in modulating the proteolytic activities of metalloproteinases, which are pivotal in tissue remodeling and especially relevant in chronic inflammatory conditions. The presence of hydrophobic residues (valine and leucine) in this peptide could synergize with its native role, suggesting a dual functionality—both as a protease inhibitor and as an anti‐inflammatory agent (Nagase and Harris Jr. 1983). Similarly, EE9 (EVSGLEQLE), a peptide originating from ovalbumin, presents an interesting profile. Given that ovalbumin is the primary protein in egg whites, the potential of EE9 might stem from the immunomodulatory properties of its parent protein, directing inflammatory responses precisely. The peptide sequence of EE9, rich in residues like glutamic acid and hydrophobic amino acids such as valine and leucine, may enhance interactions with cell membranes, possibly attenuating pro‐inflammatory cascades (Li et al. 2022). Peptide RE8 (RKLLEGEE), sourced from vimentin, a type III intermediate filament protein, also manifested a strong PreAIP. Vimentin is a crucial component of the cellular cytoskeleton, and its potential function might be linked to cell integrity and inflammatory responses (Paulin et al. 2022). The inherent properties of RE8, which include both hydrophilic and hydrophobic residues, may grant it the ability to interface with cellular machinery, thereby dampening inflammatory pathways. RP8 (RVTNLGRP), derived from integrin alpha‐6, is another peptide that merits attention. Integrins play a crucial role in cellular adhesion and signaling, contributing to diverse physiological processes. These include modulating macrophage actions during inflammation (Mercurio et al. 2001). The presence of hydrophobic residues (valine and leucine) in this peptide could suggest its role in mediating integrin‐lipid bilayer interactions, offering a possible mechanism for its anti‐inflammatory activity. Investigating peptides with medium AIP scores, we identified several intriguing sequences that could potentially contribute to the anti‐inflammatory capacities of EWH, albeit to a lesser degree compared to the higher‐scoring counterparts. For instance, LE9 (LPDEVSDLE), from ovalbumin‐related protein X, shares significant sequence homology and structural properties with ovalbumin (Rehault‐Godbert et al. 2013). This suggests potential in binding and neutralizing pro‐inflammatory agents, though further verification is required. PA11 (PAPSTGGRIVA), associated with ovocleidin‐116, plays a role in eggshell mineralization (Horvat‐Gordon et al. 2008). Its strong interactions with mineral ions might imply involvement in cellular signaling processes, which could be significant in the inflammatory response. Finally, PK8 (PEKKAKKK), from ovofactor‐1, may have roles related to its protease inhibitory origins, potentially contributing to protective mechanisms against inflammation (Nakamura et al. 1995). After a detailed peptidomic analysis, we explored the anti‐inflammatory potential of seven peptides in LPS‐induced RAW 264.7 macrophages, gaining critical insights. LPS, found in the outer membrane of gram‐negative bacteria, is a well‐acknowledged potent endotoxin (Wang and Quinn 2010). When introduced to RAW 264.7 macrophages, LPS initiates a cascade of inflammatory responses, amplifying the production of inflammatory markers, with nitric oxide (NO) standing out as paramount (Forstermann and Sessa 2012). Elevated NO production is a defining feature of inflammation, often accompanying LPS stimulation and underpinning various pro‐inflammatory processes. Our data highlighted the exceptional anti‐inflammatory performance of IS8. Originating from ovostatin, IS8 demonstrated significant anti‐inflammatory activity, especially in reducing the inflammatory marker nitric oxide, aligning seamlessly with its high AIP score. This activity underscores its promise as a potent therapeutic agent. Equally commendable were PA11 and PK8. Even with their medium AIP scores, their effective roles in counteracting inflammation were evident. This efficacy, when contrasted with their preliminary AIP scores, paints a vivid picture of the multifaceted nature of peptide functionality. RP8 and LE9 also offered valuable insights. While EE9 and RE8 were characterized by high AIP scores, their exhibited anti‐inflammatory activities in RAW 264.7 macrophages were notably subdued. This highlights the inherent complexities in peptide functionalities, suggesting that an AIP score, although indicative, might not always be reflective of the peptide's actual biological performance. The anti‐inflammatory activities of IS8, PA11, and PK8 peptides resonate with prior investigations, where specific ovalbumin hydrolysates exhibited notable inhibition of NO and iNOS production in LPS‐challenged RAW 264.7 macrophages (Kim et al. 2020). As a result, IS8, PA11, and PK8 emerged as the frontrunners in this study. Their combined potential to modulate inflammation, coupled with their intriguing AIP scores, paved the way for a more in‐depth investigation into their molecular actions. Following our in vitro assays that highlighted the anti‐inflammatory potential of IS8, PA11, and PK8, qRT‐PCR was employed to gain a deeper understanding of their molecular mechanisms.

Inflammation, a crucial physiological response, relies heavily on a cascade of signaling molecules (Roe 2021). At the core of this complex response are pro‐inflammatory mediators, including key cytokines like TNF‐α and IL‐1β, and pivotal enzymes such as iNOS and COX‐2. These agents play a central role in amplifying and maintaining inflammatory responses, with their overexpression often correlated with pathogenic inflammatory conditions. Conversely, IL‐10 serves as a protective counterbalance, exerting anti‐inflammatory effects that are imperative to curbing unchecked inflammation. In our study, the overarching role of LPS in elevating the expression of pro‐inflammatory agents in RAW 264.7 macrophages was apparent, echoing findings from numerous prior research efforts. Upon LPS challenge, we observed a pronounced upregulation in the mRNA expression levels of TNF‐α, IL‐1β, iNOS, and COX‐2, establishing a pro‐inflammatory environment. Against this backdrop, the modulatory potential of bioactive peptides from EWH, particularly IS8, PA11, and PK8, was assessed. Our findings shed light on the profound impact of these peptides. They did not merely present a passive defense against LPS‐induced inflammation but actively reversed the LPS effect. Notably, IS8 and PK8 were particularly potent, significantly downregulating the mRNA expression of the aforementioned pro‐inflammatory agents. This dose‐dependent downregulation hints at their potential mechanisms of action, possibly interacting with upstream signaling pathways or molecular targets to stifle the transcription of pro‐inflammatory genes. Positioning our findings within a broader research landscape, similar anti‐inflammatory trends were highlighted in earlier studies on egg white derivatives. For instance, Choi et al. (2013) and Jahandideh et al. (2017) accentuated the capacity of egg white derivatives to diminish the synthesis of certain inflammatory agents (Choi et al. 2013). Their findings resonate with ours, collectively confirming the perception of egg white derivatives as powerful modulators of inflammatory pathways. However, our observations took a further leap with the evident upsurge in IL‐10 expression in peptide‐treated macrophages. This cytokine, often celebrated for its anti‐inflammatory ability, offers a protective counter to pro‐inflammatory effects. The heightened IL‐10 levels in the presence of IS8, PA11, and PK8 illustrate these peptides' harmonized approach to inflammation modulation, combining the suppression of pro‐inflammatory signals with the amplification of anti‐inflammatory responses. Following our qRT‐PCR results, extensive research on egg‐derived peptides and their role in modulating inflammation and related pathways offers insightful parallels. A particularly interesting discovery was made with the low‐molecular‐weight fractions of alcalase hydrolyzed egg ovomucin, which effectively quelled inflammatory activity in human dermal fibroblasts by targeting the TNF‐mediated nuclear factor κB pathway (Sun et al. 2016). Another compelling study on ovotransferrin and its di‐peptides spotlighted their anti‐inflammatory properties in TNF‐α‐induced Caco‐2 cells (Wang et al. 2017). Remarkably, the egg‐derived tripeptide (IRW) emerged as a potent inhibitor of LPS‐induced osteoclastogenesis and inflammatory bone resorption in RAW 264.7 macrophages, revealing the breadth of application for egg‐derived peptides (Shang and Wu 2020). On the vascular front, the same tripeptide, IRW, demonstrated potential in counteracting vascular remodeling by suppressing Ang II‐stimulated migration of vascular smooth muscle cells (Liao et al. 2018). Lastly, broadening the scope beyond peptides, lysozyme's unique capability to transcriptionally regulate the TNF‐α pathway genes in monocytes added another layer to our understanding of egg protein's anti‐inflammatory potential (Bergamo et al. 2019).

Molecular docking analysis provided compelling insights into the interaction between iNOS, a pivotal enzyme in inflammation, and bioactive peptides derived from EWH. iNOS, or inducible nitric oxide synthase, plays a central role in the inflammatory response by catalyzing the production of NO from L‐arginine. While NO is an essential signaling molecule under normal physiological conditions, its overproduction during inflammation contributes to oxidative stress and tissue damage, making iNOS a key target in the management of inflammatory diseases (Cinelli et al. 2020). The docking scores indicated that all tested peptides—IS8, PA11, and PK8—exhibited stronger binding affinities to the iNOS active site compared to the co‐crystallized ligand AT2, a well‐characterized inhibitor of iNOS. Among the peptides, PK8 and IS8 showed the highest docking scores, followed closely by PA11, which scored moderately. This enhanced binding potential can be attributed to their ability to form stabilizing interactions such as hydrogen bonds, hydrophobic contacts, and π‐π stacking with key residues in the iNOS binding pocket (Suaifan et al. 2012).

## 5 Conclusion

This study comprehensively investigated the anti‐inflammatory potential of EWH and its constituent peptides. Peptidomic analysis identified peptides with varying Anti‐Inflammatory Potential (AIP) scores, which displayed significant efficacy against LPS‐induced inflammation in RAW 264.7 macrophages. Molecular docking studies revealed that peptides IS8, PA11, and PK8 exhibited strong binding affinities to the iNOS active site, suggesting their potential interference with its catalytic function and reduction of NO production, a key pro‐inflammatory mediator. These peptides demonstrated dual functionality by suppressing pro‐inflammatory mediators while enhancing anti‐inflammatory responses. Our findings establish EWH and its bioactive peptides as promising candidates for anti‐inflammatory interventions. Future studies should focus on clinical applications and the development of therapeutic strategies based on these peptides.

## Author Contributions

**Ruedeemars Yubolphan:** data curation (equal), investigation (equal), methodology (equal), visualization (equal), writing – original draft (equal), writing – review and editing (equal). **Kajhonpan Phongsiri:** data curation (equal), formal analysis (equal), investigation (equal), visualization (equal). **Nanthakan Mongkhammee:** data curation (equal), formal analysis (equal), investigation (equal), visualization (equal). **Jutamas Pidech:** formal analysis (equal), investigation (equal), visualization (equal). **Sittiruk Roytrakul:** formal analysis (equal), investigation (equal), methodology (equal), resources (equal), writing – review and editing (equal). **Chonticha Suwattanasophon:** investigation (equal), methodology (equal), visualization (equal), writing – review and editing (equal). **Kiattawee Choowongkomon:** methodology (equal), resources (equal), supervision (equal), writing – review and editing (equal). **Sakda Daduang:** methodology (equal), resources (equal), supervision (equal), writing – review and editing (equal). **Watcharee Khunkitti:** methodology (equal), resources (equal), supervision (equal), writing – review and editing (equal). **Nisachon Jangpromma:** conceptualization (equal), data curation (equal), funding acquisition (equal), methodology (equal), project administration (equal), resources (equal), supervision (equal), visualization (equal), writing – review and editing (equal).

## Conflicts of Interest

The authors declare no conflicts of interest.

## Data Availability

The data that support the findings of this study are available on request from the corresponding author. The data are not publicly available due to privacy or ethical restrictions.
